# Venovenous Extracorporeal Membrane Oxygenation as a Treatment for Obesity Hypoventilation Syndrome

**DOI:** 10.1155/2017/9437452

**Published:** 2017-02-23

**Authors:** Nao Umei, Shingo Ichiba

**Affiliations:** Department of Surgical Intensive Care Medicine, Nippon Medical School Hospital, Tokyo, Japan

## Abstract

The mortality rate for respiratory failure resulting from obesity hypoventilation syndrome is high if it requires ventilator management. We describe a case of severe acute respiratory failure resulting from obesity hypoventilation syndrome (BMI, 60.2 kg/m^2^) successfully treated with venovenous extracorporeal membrane oxygenation (VV-ECMO). During ECMO management, a mucus plug was removed by bronchoscopy daily and 18 L of water was removed using diuretics, resulting in weight loss of 24 kg. The patient was weaned from ECMO on day 5, extubated on day 16, and discharged on day 21. The fundamental treatment for obesity hypoventilation syndrome in morbidly obese patients is weight loss. VV-ECMO can be used for respiratory support until weight loss has been achieved.

## 1. Introduction

Obesity hypoventilation syndrome (OHS) is defined as the combined presence of obesity (BMI > 30 kg/m^2^) and hypoventilation (partial pressure of arterial carbon dioxide [PaCO_2_] > 45 mmHg) in the absence of other diseases that contribute to hypoventilation [1–4]. No drugs that effectively treat OHS are currently available; the only effective treatment is weight loss [2, 3]. However, weight loss is slow to occur, and patients with OHS experience rapid deterioration until respiratory failure develops. In these patients, noninvasive positive-pressure ventilation or invasive mechanical ventilation may be effective strategies [2–4].

The use of venovenous extracorporeal membrane oxygenation (VV-ECMO) has increased as a bridge to recovery for severe acute respiratory failure (ARF) refractory to conventional support [5]. However, few studies have reported successful outcomes for patients with OHS with ARF requiring VV-ECMO. We herein report a case of severe ARF resulting from OHS in a morbidly obese patient for whom VV-ECMO was therapeutic.

## 2. Case Presentation

A male patient in his mid-40s with a BMI of 60.2 kg/m^2^ presented to a local hospital to undergo upper gastrointestinal endoscopy for epigastralgia. The patient's oxygen saturation was approximately 80%, and he was admitted to the general ward. His condition worsened, and blood gas analysis revealed the following: pH, 7.093; PaCO_2_, 115.3 mmHg; and PaO_2_, 96.7 mmHg on 100% oxygen. He was intubated and mechanically ventilated. His primary physician performed Swan–Ganz catheterization, which revealed the following: pulmonary capillary wedge pressure, 19 mmHg; pulmonary artery pressure, 51/28 (36) mmHg; and cardiac index, 4.78 L/min/m^2^. Echocardiography revealed an ejection fraction of 45.7% and tricuspid regurgitation pressure gradient of 29 mmHg without evidence of organic valvular disease. Chest radiographs showed bilateral pulmonary edema and consolidation. The diagnosis was ARF resulting from OHS combined with cardiac failure and pulmonary hypertension. The patient's relatively long, untreated clinical course, atelectasis, and hydrostatic pulmonary edema might have developed before his admission. After 9 days of conventional therapy, his respiratory function showed no improvement; therefore, the patient was transferred to our intensive care unit. On admission, he was sedated with a respiratory rate of 39 breaths/minute, heart rate of 96 beats/minute, and blood pressure of 124/66 mmHg. The patient required a driving positive pressure of 15 cm H_2_O to generate a 400 mL (6 mL/kg) tidal volume. The fraction of inspired oxygen was 1.0; arterial oxygen partial pressure (PaO_2_) was 69.8 mmHg; PaCO_2_ was 52.1 mmHg; and pH was 7.383. Chest X-ray showed an enlarged cardiac silhouette and diffuse pulmonary infiltrates suggesting congestive heart failure and atelectasis. Given the patient's age and single-system organ failure, VV-ECMO was emergently instituted. Cannulation was performed via the right internal jugular vein with 25-French drainage cannulae (Bio-Medicus®; Medtronic, Minneapolis, MN, USA) for access and via the right femoral vein with 19-French cannulae (Medtronic) for return. The ECMO circuit was an adult ECMO bypass custom tubing pack consisting of a Rotaflow® centrifugal pump (MAQUET Cardiopulmonary GmbH, Germany) and two gas exchangers (MERA NHP Excelung NSH-R HPO-23WH-C®; Senko Medical Inc., Tokyo, Japan). We used two oxygenators in parallel to enlarge the membrane surface area. ECMO was initiated at a blood flow of 5.7 L/min and sweep gas flow of 5.0 L/min in 100% oxygen per oxygenator. The pre- and postoxygenator pressures were monitored continuously throughout the ECMO run. The lungs were rested with pressure-controlled ventilation at an inspiratory pressure of 5 cm H_2_O, positive end-expiratory pressure (PEEP) of 10 cm H_2_O, and FIO_2_ of 0.3 to 0.6 during the ECMO run, permitting tidal volumes of 100 mL support. The patient was heparinized to maintain an activated partial thromboplastin time of 50 to 80 seconds. Because of clinical signs of pulmonary congestion, diuretic and inotropic therapies (milrinone and dobutamine) were initiated, with some improvement in the hemodynamic parameters. After initiation of ECMO, the patient's hemodynamic status was stabilized and he was successfully kept in a negative hydration balance every day. Furosemide was administered at 0.1 mg/kg/min, and a negative fluid balance of −17946 mL was achieved during ECMO. Maintaining this negative balance, his body weight decreased from 167 to 143 kg and his pulmonary congestion improved with a tricuspid regurgitation pressure gradient of 17 mmHg. We also performed bronchoscopy and removed sputum daily, which improved atelectasis, compliance, and oxygenation.

On day 5, we performed a 2-hour ECMO off-trial. The patient tolerated the trial without O_2_ flow to the oxygenator; therefore, we decided to wean him off ECMO. After weaning off ECMO, his respiratory condition worsened without a high PEEP, and his PaO_2_ decreased; therefore, we weaned PEEP slowly. We removed the mechanical ventilation 11 days later. Using a 2 L/min nasal cannula, the blood gas analysis results were as follows: pH, 7.415; PO_2_, 115 mmHg; and PCO_2_, 39.8 mmHg. The patient was discharged from the hospital 5 days after extubation with a final body weight of 125.2 kg.

## 3. Discussion

Many studies have shown increased morbidity and mortality in obese patients in the intensive care unit [6–8]. Obesity is associated with a radically increased risk of diabetes and other serious cardiovascular and pulmonary diseases as well as an increased risk of death from these disorders. Respiratory disorders such as OHS and pulmonary emboli are a frequent cause of sudden death [8]. The mechanism by which obesity leads to hypoventilation is not certain. Resistance to leptin, which is produced by fat cells, may be associated with hypoventilation [9, 10]. No drugs that effectively treat OHS are currently available; weight loss is the only effective treatment measure [2, 3].

Ull et al. [11] reported two cases involving obese patients with postoperative ARF treated with VV-ECMO. The first patient, who had a BMI of 77 kg/m^2^, developed severe ARF on the fourth day of postsurgical intervention, and VV-ECMO was performed [11]. The second patient, who had a BMI of 88.6 kg/m^2^, developed severe ARF because of sepsis after treatment of necrotizing fasciitis, and VV-ECMO was indicated [11]. Extreme obesity is not a contraindication for VV-ECMO implantation [12–14]. However, treating extremely obese patients with VV-ECMO poses a special challenge for intensivists. Femorojugular venovenous percutaneous cannulation is associated with several pitfalls including difficulty locating the anatomical landmarks and the inability to visualize the blood vessels sonographically [14]. The cannulation procedure must be performed by experienced practitioners. We stretched the extensive slack of the patient's skin using adhesive tape to expose the femoral and neck regions for cannulation. In addition, we inserted cushions underneath the lower back and shoulder region so that the cannulation procedure was feasible by bending the body backward. Theoretically, increased cardiac output associated with higher body weight may surpass the oxygenation capacity of the ordinary ECMO system. Additionally, CO_2_ elimination through the native lungs decreases in obese patients. In such cases, two oxygenators are necessary for VV-ECMO. Applying two oxygenators in parallel diverts the circuit blood flow into two oxygenators, which reduces the blood flow through each oxygenator, resulting in much more efficient oxygenation. Moreover, the total surface area of the membrane oxygenator is doubled, increasing the total amount of CO_2_ removal through the VV-ECMO system.

The primary treatments in our case involved controlling the patient's water balance to a negative balance and performing daily bronchoscopy. The use of furosemide resulted in adequate weight loss, which is the indicated treatment for OHS. We believed that we could successfully manage his water balance because VV-ECMO stabilized his hemodynamic status. During VV-ECMO, we achieved lung rest by reducing the ventilatory pressure, which reduced the intrathoracic pressure and increased the left and right cardiac output. During VV-ECMO, oxygenated blood provided from the ECMO system mixes with deoxygenated blood from the native circulation in the right atria, and preoxygenated blood then travels to the pulmonary circulation. This oxygenated blood in the pulmonary circulation leads to pulmonary vascular dilatation, which in turns leads to decreased pulmonary vascular resistance, reduces septal displacement, and improves left ventricular function [15, 16].

This allowed us to remove approximately 18 L of water, which treated his original obesity-related pulmonary hypertension. The secondary treatment in our case was daily bronchoscopy. We were able to perform bronchoscopy safely because the patient's oxygenation was controlled by the VV-ECMO. Frequent bronchoscopy during ECMO is required for bronchial toilet and to remove sputum that can cause atelectasis.

The main limitation of this case report is that a pulmonary arterial catheter could not be used because of the high risk induced by extreme obesity; therefore, the pulmonary arterial pressure and cardiac output could not be measured directly. However, based on its physiological effects, we believe that VV-ECMO effectively reduced the intrathoracic pressure and pulmonary vascular resistance, which stabilized our patient's hemodynamic status and permitted the removal of large volumes of water from the body. Miranda et al. reported that initiation of VV-ECMO decreased the mean pulmonary artery pressure and increased the cardiac index and mixed venous oxygenation [15]. Additionally, the decrease in the PaCO_2_ after the start of VV-ECMO contributed to the decrease in the mean pulmonary artery pressure [16].

Obese patients are viewed as more complex to manage by ECMO. However, it is possible to use VV-ECMO for life-threatening respiratory failure if percutaneous cannulation is possible. The use of two membranes is also an important strategy for obese patients.

In conclusion, VV-ECMO is a useful modality for managing severe ARF resulting from OHS because it may enable sufficient gas exchange and improve pulmonary circulation by reduction of pulmonary vascular resistance until diuretic weight loss is achieved.
